# High-Throughput Identification of the *Rhodnius prolixus* Midgut Proteome Unravels a Sophisticated Hematophagic Machinery

**DOI:** 10.3390/proteomes8030016

**Published:** 2020-07-24

**Authors:** Radouane Ouali, Karen Caroline Valentim de Brito, Didier Salmon, Sabrina Bousbata

**Affiliations:** 1 Proteomic Plateform, Laboratory of Microbiology, Department of Molecular Biology, Université Libre de Bruxelles, 6041 Gosselies, Belgium; radouane.ouali@ulb.be; 2 Institute of Medical Biochemistry Leopoldo de Meis, Centro de Ciências e da Saúde, Federal University of Rio de Janeiro, Rio de Janeiro RJ 21941-902, Brazil; karen.brito@bioqmed.ufrj.br (K.C.V.d.B.); salmon@bioqmed.ufrj.br (D.S.)

**Keywords:** Chagas disease, hematophagy, *Rhodnius*, midgut, proteomics

## Abstract

Chagas disease is one of the most common parasitic infections in Latin America, which is transmitted by hematophagous triatomine bugs, of which *Rhodnius prolixus* is the vector prototype for the study of this disease. The protozoan parasite *Trypanosoma cruzi*, the etiologic agent of this disease, is transmitted by the vector to humans through the bite wound or mucosa. The passage of the parasite through the digestive tract of its vector constitutes a key step in its developmental cycle. Herewith, by a using high-throughput proteomic tool in order to characterize the midgut proteome of *R. prolixus*, we describe a set of functional groups of proteins, as well as the biological processes in which they are involved. This is the first proteomic analysis showing an elaborated hematophagy machinery involved in the digestion of blood, among which, several families of proteases have been characterized. The evaluation of the activity of cathepsin D proteases in the anterior part of the digestive tract of the insect suggested the existence of a proteolytic activity within this compartment, suggesting that digestion occurs early in this compartment. Moreover, several heat shock proteins, blood clotting inhibitors, and a powerful antioxidant enzyme machinery against reactive oxygen species (ROS) and cell detoxification have been identified. Highlighting the complexity and importance of the digestive physiology of insects could be a starting point for the selection of new targets for innovative control strategies of Chagas disease.

## 1. Introduction

Hematophagous insects are ectoparasites with a wide range of hosts from which they take blood. The blood meal satisfies their nutritional requirements and allows their further development [1]. Most hematophagous insects are vectors of human diseases caused by pathogens [2]. Chagas disease or American trypanosomiasis is one of the most common parasitic infections, which is transmitted to humans by a large group of bloodsucking bugs [3]. According to WHO, the latest available estimates indicate that this parasitosis affects about 8 million people, with, in 2017, 14,000 deaths [4]. The causative agent of this disease, the protozoan parasite *Trypanosoma cruzi*, is transmitted by hematophagous triatomine bugs (Hemiptera, Reduviidae, and Triatominae) [5]. *Rhodnius prolixus* is one of the most important vector species in the transmission of *T. cruzi* to humans and the prototype for the study of Chagas disease transmission [3]. 

A blood meal is necessary for *R. prolixus*, not only to complete its developmental cycle through five nymphal stages before becoming an adult but, also, for egg laying [6,7]. The life cycle of *T. cruzi* begins when the triatomine takes its blood meal from an infected host by ingesting the bloodstream trypomastigote forms. Once in the midgut of the insect, the parasites transform into the epimastigote form, which adheres to the perimicrovillar membrane lining intestinal cells, and multiply by binary fission. The epimastigotes then migrate to the hindgut (rectum) and are further transformed into infective metacyclic trypomastigotes, which are excreted by the insect via feces and urine [5,8]. Upon insects feeding on the blood of the host, the contaminated feces and urine are released on the vertebrate host skin and, subsequently, enter the bloodstream when scratching the bite wound or via the mucosa [9].

The midgut plays a major role in the transmission of the disease, as it is both the primary site for digestion of the blood meal and interaction with the parasites. In addition, *T. cruzi* development inside the digestive tract of *R. prolixus* is a crucial step in its life cycle [10]. Indeed, the midgut, which ensures the digestion of large amounts of blood, is also the site through which the parasite transforms to the infectious metacyclic trypomastigote form (named metacyclogenesis) [11]. Upon blood feeding, the insect ingests 6–12 times its weight of blood, which is stored in the anterior part of the midgut (AM) [12]. Subsequently, after the lysis of erythrocytes, the content of the AM is released in the posterior midgut (PM) where the blood is digested, and heme is detoxified to generate large amounts of crystalline hemoglobin (hemozoin). Finally, in the hindgut (H), urate crystals accumulate, and blood remnants are excreted [13]. Blood feeding induces drastic changes in the triatomine physiology, and the migration of the parasite through its digestive tract exposes it to various stressful factors, such as changes in temperature, osmolarity, pH, and oxidative and proteolytic stresses [12].

A recent transcriptome analysis of the *R. prolixus* digestive tract has made it possible to characterize genes whose transcription is altered after a blood meal [14]. This work allowed the description of major groups of proteins implicated in key functions in this tissue. However, as gene expression is highly regulated at different levels (i.e., transcription initiation but, also, post-transcriptional and translational), it is not always correlated with protein abundance [15,16]. In the present study, the whole midgut proteome of *R. prolixus* in the early hours after blood ingestion was characterized. The aim of this work is to validate at the protein level the biological information already obtained from the transcriptome of the digestif tract. Moreover, the functional identification of proteins from the midgut should provide a better understanding of the physiology of the insect.

## 2. Materials and Methods 

### 2.1. Biological Material 

The insects are kept at a temperature of 28 °C and 60–80% humidity, under a photoperiod of 12 h light/12 h dark. Insects were fed 3 weeks after the last meal with rabbit blood. Permission for experimental use of insects was obtained by the Federal University of Rio de Janeiro (Registry #IBQM001).

### 2.2. Dissection of the Insect and Preparation of the Digestive Tract

For the starved insects, the digestive tract was taken 3 weeks after the first meal, and for the blood-fed insects, the digestive tract was dissected 6 h after blood ingestion. Three biological replicates per condition, each of five randomly selected insects, were used. Insects were dissected in cold phosphate-buffered saline solution (0.15-M NaCl, 43-mM Na_2_HPO_4_, 1.4-mM KH_2_PO_4_, and 2.7-mM KCl, pH 7.4), and the midgut, carefully pulled apart, was separated in AM and PM compartments. AM and PM were incised and rigorously washed with cold phosphate-buffered saline to remove digestive juices. Intact midguts were stored in STE buffer (0.1-M Tris, 0.05-M NaCl, and 0.05-M EDTA (Ethylenediaminetetraacetic acid), pH 7.4) at −80 °C until use. Samples were processed under strict cold conditions to avoid protein degradation as proteases were omitted from the sample buffer, which is required for enzymatic activity assays. 

### 2.3. Proteins Extraction

Freezing/thawing at −80 °C in the storage buffer was sufficient to recover AM and PM soluble proteins from the supernatant by centrifugation at 13,000× *g* for 15 min at 4 °C. Protein concentration was determined with Pierce 660 nm Protein Assay (Thermo Scientific Inc., Rockford, IL, USA) using a series of bovine serum albumin (BSA) solutions as the protein concentration standards. Starved and blood-fed biological replicates were processed separately throughout the whole analyses.

### 2.4. Sample Preparation Prior to Mass Spectrometry

Ten micrograms of proteins from three biological replicates of the starved and blood-fed conditions were mixed with lysis buffer (20-mM HEPES (4-(2-hydroxyethyl)-1-piperazineethanesulfonic acid), 8-M urea, and 0.5-M dithiothreitol, pH 8), and the solution was mixed and incubated at 55 °C for 30 min. Then, 0.5 M of iodoacetamide were added, and the sample was incubated for 15 min at room temperature in the dark. The samples were diluted 2-fold with 20-mM HEPES, pH 8 prior to enzymatic digestion. The samples were digested first by 1 µg of LysC (Promega, Leiden, Netherlands) for 4 h at 37 °C, then by 1 µg of trypsin (Promega, Leiden, Netherlands) overnight at 37 °C. Digestion was stopped by adding 1% trifluoroacetic acid. The resulting peptide mixture was purified using OMIX C_18_ pipette tips (Agilent, Santa Clara, CA, USA). The purified peptides were dried completely and resuspended in 20-µL loading solvent (0.1% TFA (**Trifluoroacetic acid)** in water/acetonitrile (2:98 *v/v*))—of which, 5 µL corresponding to 3 µg were injected for LC-MS/MS analysis on an Ultimate 3000 RSLC nano ProFlow system on-line connected to a Q-Exactive HF mass spectrometer (Thermo, Waltham, MA, USA). Trapping was performed at 10 μL/min for 4 min in loading solvent A on a 20-mm trapping column (made in-house; 100-μm internal diameter (I.D.) and 5-μm beads, C_18_ Reprosil-HD, Dr. Maisch, Ammerbuch, Germany), and the sample was loaded on a 40-cm analytical column (made in-house; 75-μm I.D. and 1.9-μm beads, C_18_ Reprosil-HD, Dr. Maisch, Ammerbuch, Germany) kept at a constant temperature of 50 °C. Peptides were eluted at a constant flow rate of 300 nL/min by a nonlinear gradient reaching 56% solvent B (0.1% of formic acidin water/acetonitrile (2:8, *v/v*)) in 87 min. The mass spectrometer was operated in data-dependent mode, automatically switching between MS and MS/MS acquisition for the 12 most abundant ion peaks per MS spectrum. Full-scan MS spectra (375–1500 *m/z*) were acquired at a resolution of 60,000 in the orbitrap analyzer after accumulation to a target value of 1 × 10^5^. The 12 most intense ions above a threshold value of 1.3 × 10^4^ were isolated for fragmentation at a normalized collision energy of 30% after filling the trap at a target value of 1× 10^3^ for a maximum of 80 ms. MS/MS spectra (200–2000 *m/z*) were acquired at a resolution of 15,000 in the orbitrap analyzer.

### 2.5. Mass Spectrometric Data Analyzes

Data analysis was performed with MaxQuant software (version 1.6.3.4, Max Planck Institute of Biochemistry, Munich, Germany) using the Andromeda search engine with default search settings, including a false discovery rate set at 1% on both the peptide and protein level. Spectra were searched against *R. prolixus* (UniProt Tax ID: 13249) proteins in the UniProt/Swiss-Prot reference database (UniProt Proteome ID: UP000015103). The mass tolerance was set to 20 ppm for peptide masses during the first-round Andromeda search, and 4.5 ppm was set for the peptide mass tolerance during the main search. Enzyme specificity was set to the C-terminal to arginine and lysine, also allowing cleavage at arginine/lysine-proline bonds, with a maximum of two missed cleavages. Variable modifications were set to the oxidation of methionine (to sulfoxides) and acetylation of the protein N-termini. A minimum of one unique peptide in total was required for identification. We allowed for matching between runs using a 1.5-min match time window and a 20-min alignment time window. Proteins were quantified by the MaxLFQ algorithm integrated in the MaxQuant software. A minimum ratio count of two unique or razor peptides was required for quantification. 

Further data analysis was performed with the Perseus software (version 1.6.2.1, Max Planck Institute of Biochemistry, Munich, Germany) after loading the protein groups file obtained previously by MaxQuant software. First, proteins identified by site and reverse database hits were removed. Data from three biological replicates of both starved and 6-h post-fed samples were grouped as two different conditions (starved and blood-fed), and proteins with less than 3 valid values in at least one condition were removed. Then, missing values from the other condition were imputed with values from the lower part of the normal distribution representing the detection limit. A protein list generated by Perseus software containing the proteomic identification parameters (unique peptides, sequence coverage percentage, and identification score) was then created. The raw data, as well as the files generated by MaxQuant and Perseus, are available on ProteomeXchange Consortium (PXD019150) and MassIVE repository (MSV000085406).

### 2.6. Functional Characterization and Protein Classification

UniProt ID numbers from the protein list generated by Perseus were searched against UniProtKB using the Retrieve/ID mapping tool (https://www.uniprot.org/uploadlists). This allowed us to associate the UniProt accession with the corresponding protein names, gene ontology categories and their IDs, molecular functions, protein families, subcellular locations, biological processes, signal peptides, molecular weight (MW), post-translational modifications, and VectorBase IDs. Protein classification was then performed according to Gene Ontology (GO) hierarchy, using PANTHER (Protein ANalysis THrough Evolutionary Relationships) classification system (http://www.pantherdb.org/) [17]. Proteins of interest for hematophagy in Table 1 were searched in the transcriptome (Supplementary Materials Table S2) of the digestive tract of *R. prolixus* by Ribeiro et al. [14], and the number of transcripts reads was reported in Table S2. 

### 2.7. Cathepsin D Activity Assay

The fluorogenic substrate (Bz-Arg-Gly-Phe-Phe-Pro-4-Methoxy-2-naphthylamide, Sigma, Overijse, Belgium) was prepared following the supplier’s instructions. The assay mixture contained 100 μL of 40 μM of substrate prepared in citrate phosphate buffer at pH 5.2. Protein samples that contain cathepsin-D will cleave the synthetic substrate to release fluorescence, which can then be quantified using a fluorescence plate reader. Fifteen micrograms of *R. prolixus* AM and PM protein extracts from 6-h post-fed insects were added to the mixture. For the activity assay in the presence of the inhibitor, 15 µg of the extract was preincubated in the assay buffer, with 10 μM of pepstatin A (Sigma, Overijse, Belgium). Proteolytic activity was continuously measured for 90 min after addition of the substrate in a fluorescence reader (SpectraMax i3, Molecular Devices, San Jose, CA, USA) at 340-nm excitation and 425-nm emission wavelengths [18]. The assay was performed using three biological replicates, and the enzyme activity curve was made from the average of relative fluorescence units of the three biological replicates, and their standard deviations are represented by error bars.

## 3. Results

### 3.1. Protein Identification and Annotation

**Figure 1:**
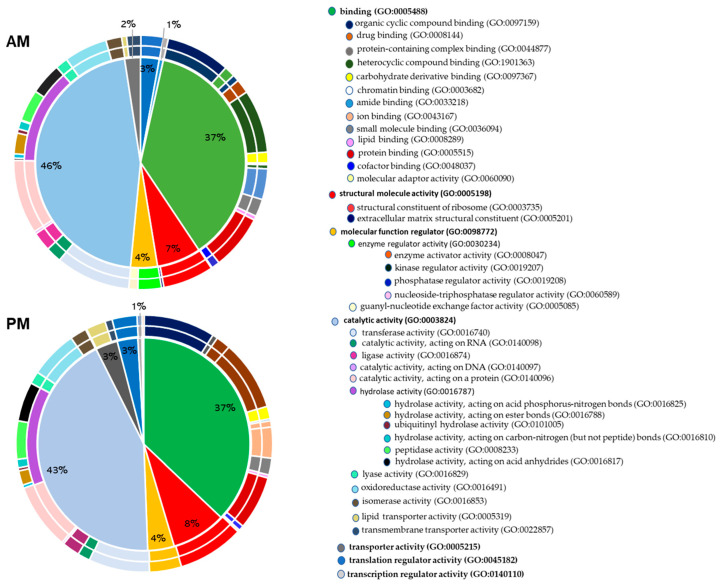
Pie charts of the molecular functions of the identified proteins by high-throughput LC-MS/MS in the anterior midgut (AM) and posterior midgut (PM) according to Gene Ontology classification. The percentage in each category is calculated based on the ratio between the number of proteins in each category to the corresponding total proteins identified in each gut tissues. Protein categories in the right panel are listed from the pie chart clockwise and starting with proteins implicated in binding. The corresponding Gene Ontology (GO) numbers are indicated between the brackets.

**Figure 2:**
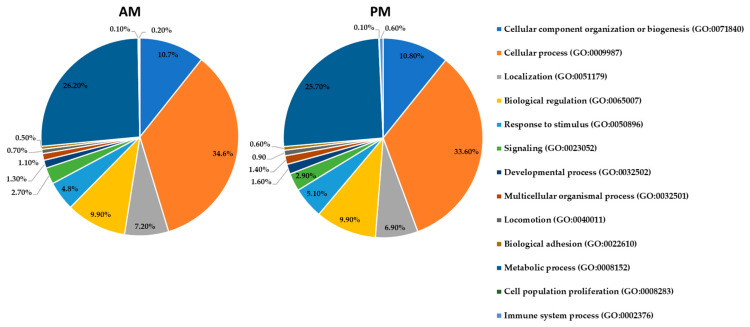
Pie charts of the biological functions of the identified proteins by high-throughput LC-MS/MS in the AM and PM according to the Gene Ontology classification. The percentage in each category is calculated based on the ratio between the number of proteins in each category to the corresponding total proteins identified in each gut tissue. Protein categories in the right panel are listed from the pie chart clockwise and starting with proteins implicated in cellular component organization. The corresponding GO numbers are indicated between the brackets.

This proteomic study of *R. prolixus* midgut using shotgun technology allowed the identification of 1471 proteins in the AM and 1132 proteins in the PM, which were identified with at least two or more unique peptides. This corresponds to about 11% of the insect’s gene products from the genome of *R. prolixus*, which predicted 15,456 putative genes coding for proteins [19]. It is worth mentioning that the number of proteins identified in the AM is higher than that in the PM. The complete list of proteins identified in the AM and PM is provided in Table S1 and classified according to their molecular functions (Figure 1) and biological processes (Figure 2), as recovered from UniProt using the Retrieve/ID mapping tool (https://www.uniprot.org/uploadlists).

Figure 1 shows that the most abundant category of the proteins identified according to their molecular function are those that have a catalytic function (46% in the AM and 43% in the PM), with hydrolases as the most represented enzymatic class (13% in the AM and 14% in the PM). This category is followed by proteins with binding activities representing 37% in both the AM and PM, structural functions (7% in the AM and 8% in the PM), regulators (4% in the AM and PM), transporters (2% in the AM and 3% in the PM), and translation and transcription regulators (3% in the AM and 1% in the PM). These same functional categories have also been identified in other insect midgut proteomes, such as the Glossina palpalis gambiensis tsetse fly [20] and in ticks Ornithodoros moubata [21] and Ornithodoros erraticus [22]. As observed in other proteomics studies [23], proteins of unknown functions are highly represented, with 35% of all proteins identified in the AM and 32% of those identified in the PM. As argued by Wood et al., 2019, research on these unstudied proteins will lead to the discovery of new biological functions that might outweigh the considerable efforts made on familiar genes [23].

At the biological level, Figure 2 shows that the most abundant categories are cellular processes (35% in the AM and 34% in the PM), metabolic processes (26% in both the AM and PM), and cellular component organization or biogenesis (11% in the AM and PM). The other minor biological processes such as cell signaling, cell proliferation, and locomotion range from 0.5–10% in the two gut tissues. Hence, most of the identified proteins in this work have a catalytic activity that appears to be vital for the insect, such as those involved in the energy-related metabolism of blood meal proteins in *R. prolixus*. Therefore, proteins involved in this physiologic pathway have been further investigated to unravel the complete hematophagic machinery of *R. prolixus.*

### 3.2. Blood Uptake and Digestive Machinery

Triatomines are blood-sucking insects that ingest large amounts of blood to ensure their development from first instar nymphs to the adult stage, in order to resist to a long period of starvation and for oviposition. To guarantee the ingestion and processing of a large quantity of blood, *R. prolixus* has developed a sophisticated machinery to ensure its supply. We have paid particular attention to proteins involved in the ingestion and processing of blood (Table 1).

#### 3.2.1. Heat Shock Proteins (HSPs)

Heat shock induces diverse behavioral, biochemical, and physiological changes in hematophagous insects. The first molecular response to heat stress is increasing the expression of HSPs [24]. HSPs have an important role in protecting cellular functions acting as chaperonins that ensure the functional folding of nascent proteins, minimizing their aggregation and assisting in the removal of denatured proteins [25].

Twelve HSPs belonging to four HSP families (Hsp90, Hsp70, Hsp60, and Hsp20) were identified in the AM and ten in the PM (Table S2). The HSPs identified in the AM are also present in the PM, while one Hsp90 (R4G8T8) and one Hsp70 (R4FLS6) are exclusive to the AM (bold in Table S2). In addition, five Hsp70 (R4FQG8, T1HJT8, T1IAR5, T1I0D9, and R4FLS6) were identified in this work (Table S2), and only two (R4FQG8 and T1HJT8) of them had their transcripts detected at a low level in the digestive tract transcriptome [14]. Moreover, two Hsp90 (R4FMH8 and R4G8T8); two Hsp60 (T1HTK4 and T1IAB6); and three Hsp20 (T1HKE2, R4G851, and T1HWW7) were identified in this proteomic study (indicated by their MW in Table S2). We have also noticed that the level of HSP transcripts does not always correlate with that of the protein expression levels. In addition, some HSPs identified in this work do not have their corresponding transcripts (marked absent in Table S2), and conversely, other HSP transcripts identified in the transcriptome work were absent from this proteome. The expression of a large number of HSPs in *R. prolixus* midgut might be correlated with the arrival of a large amount of warm blood, which induces an increase in the body temperature of the insect. Indeed, members of HSPs were shown to be overexpressed in the midgut of several hematophagous species after a blood meal such as *Aedes aegypti* [26], *Rhipicephalus microplus* [27], and *Ornithodoros erraticus* [22]. In that respect, Hsp70 is the most widely studied HSP as a response to heat and many other stresses [24,28]. Interestingly, it was reported that a Hsp70 knockdown in *R. prolixus* results into a significant alteration in the physiological responses to a blood meal [29]. In this case, the insects die prematurely after blood feeding after showing an alteration in the blood digestion process and a reduction in energy metabolism and immune response [29]. This suggests that these proteins are necessary not only for responding to heat stress but, also, for triggering blood digestion, probably by activating the corresponding signaling pathways. 

#### 3.2.2. Protease Inhibitors

All hematophagous invertebrates studied to date produce at least one inhibitor of coagulation to prevent blood clotting [30]. In triatomines, several protease inhibitors were identified in both saliva and in digestive tract, such as triabin from *Triatoma pallidipennis* saliva [31], infestin from *Triatoma infestans* midgut [32], and brasiliensin from *Triatoma brasiliensis* intestins [33]. The *R. prolixus* digestive tract transcriptome showed that eleven protease inhibitors with a conserved Kazal domain are transcribed in this tissue, and some of them have multiple Kazal domains [14]. Several of these products have been characterized and studied at the structural level, such as rhodniin, which is a highly specific serine protease inhibitor of thrombin through two Kazal-type domains binding [34]. In this proteomic study, we identified six protease inhibitors in the AM, three of them being shared with the PM (Table S2). Two of the protease inhibitors (T1IGD5 and R4G7P1) identified in the AM are also the most abundant transcripts of the gut (Table S2). Interestingly, three of the serine protease inhibitors found in the AM (T1HGL4, T1HUX6, and Q06684) have not been identified in the gut trancriptome of *R. prolixus* (marked absent in Table S2). The discovery of a novel Kunitz-type serine protease inhibitor may have potential as an anticoagulant drug or vaccine [35]. 

Protease inhibitors are believed to be expressed in the midgut of *R. prolixus* due to their importance in maintaining blood fluidity in the insect digestive tract by inhibiting thrombin activity, which is involved in blocking the coagulation cascade [36]. This might explain the higher number of isoforms in the AM where blood is stored. The expression of these proteins in the midgut could also play a role in the interaction with pathogens brought by the blood meal. Indeed, an overexpression of *R. prolixus* trypsin inhibitor RpTI has been observed in the AM following its infection with *T. cruzi* [37]. Interestingly, a reduction of the expression of this protein by knockdown showed a significant decrease in the *T. cruzi* load, while the bacterial load was higher compared to the control insects. The authors proposed that the modulation of the expression of RpTI is induced by *T. cruzi* to control the insect microbiota. 

#### 3.2.3. Proteases

**Figure 3:**
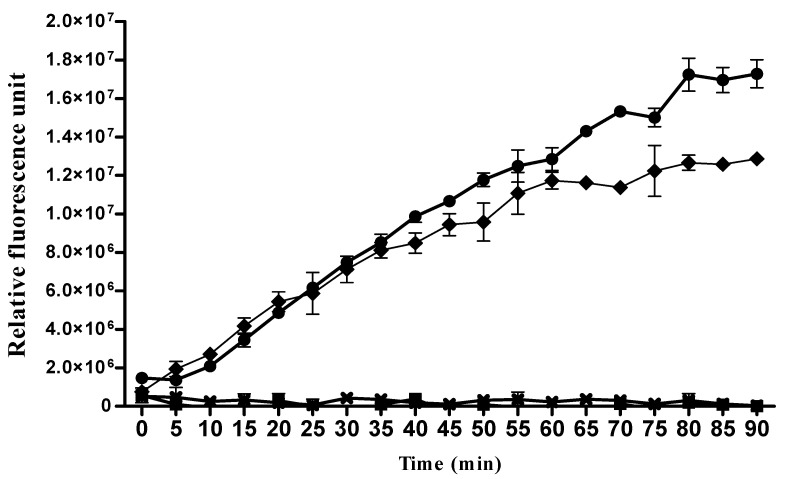
In vitro cathepsine D activity in *Rhodnius prolixus* AM and PM tissue extracts 6 h post-feeding. Cathepsin D activities in *R. prolixus* AM and PM protein extracts from 6 h post-fed insects were measured with cathepsin D-specific substrate using a continuous fluorometric assay. Activities are presented in relative fluorescence units (RFU). Cathepsin D activities of both tissues were validated by the addition of 10 μM of pepstatin A, a selective cathepsin D inhibitor. (-●-) AM, (-♦-) PM, (-■-) AM + pepstatin A, and (-**×**-) PM + pepstatin A.

Proteases are important proteins in the digestive tract due to their involvement in the digestion of hemoglobin, a major blood constituent. We identified in this work 27 proteins with proteasic activity in the AM and 29 in the PM (Table S2). The transcriptome of the digestive tract of *R. prolixus* by Ribeiro et al., 2014 [14], revealed the presence of fifteen transcribed aspartyl proteases (cathepsins D)—among which, eight of them were confirmed to be expressed at the protein level by the present study, and two of them (T1I913 and T1IEM8) are the most abundant transcripts of both the AM and PM, while two proteases (T1I882 and T1IAU3) are unique to this work. Interestingly, three aspartyl proteases (T1IEM8, T1HJV8, and T1IFK7) are present only in the AM while absent from the PM. Although the number of transcript isoforms of cathepsins D identified in the gut transcriptome was identical in the AM and the PM, their expression levels were undoubtedly higher in the AM. This difference may explain the absence of the less abundant transcripts (T1HJV8 and T1IFK7) from the proteome of the PM, but it does not account for the absence of T1IEM8, which number of reads is 460 (Table S2). We have also identified nine cysteinyl proteases in the PM, six of which are shared with the AM and three (R4FM70, R4FQ86, and T1H868) are unique to the PM (bold in Table S2). This pattern showing a higher number of aspartyl proteases isoforms in the AM and a higher number of cysteinyl proteases in the PM was observed in both the gut transcriptome and the present study. The other proteases identified in this work are carboxypeptidases, metalloproteases, and dipeptidases (Table S2). The digestion process in triatomines is different from the other hematophagous insects (e.g., tsetse flies) in which blood digestion is accomplished by the serine proteases, such as trypsin and chymotrypsin, due to their alkaline gut pH [38,39]. In the triatomines, digestion is ensured mainly by aspartyl and cysteinyl proteases [40,41]. Since no proteolytic digestion has been detected in the AM [42], the presence of cathepsins in this part of the midgut was suggested to be in the form of proenzymes, which are further activated in the PM [13,14]. Interestingly, a 2-DE reference map of the AM of *R. prolixus* identified four processed forms of cathepsins, suggesting that these enzymes could be maturated in the AM [43]. Hence, to validate this observation, we tested cathepsins D activities in both *R. prolixus* AM and PM protein extracts 6 h after a blood meal by using a fluorogenic substrate specific to cathepsin D at acidic pH. Figure 3 shows that both the AM and PM protein extracts are able to digest cathepsin D substrate and that the activity increases gradually within the 90-min-lapse time assayed. This activity is completely lost in the presence of cathepsin D inhibitor pepstatin A (Figure 3).

Surprisingly, AM proteins extracts from 6-h post-fed insects are not only capable of digesting cathepsin D substrate, but the activity of these enzymes in the AM is higher than that observed in the PM normalized to the same amount of proteins (Figure 3). These results suggest that the digestion of blood may begin in the AM, an organ that has long been considered as a blood storage compartment [40]. Since cathepsin D has been shown to be activated by acidic pH conditions or the action of other lysosomal peptidases [41], we have thus assayed AM protein extracts of blood-fed insects at both acidic pH 5.2 and neutral pH 7 (Figure S1). Although the enzymatic activity at acidic pH 5.2 was higher, nevertheless, a proteolytic activity was present at neutral pH 7 (Figure S1). The addition of pepstatin A, a specific cathepsin D inhibitor, to the AM extracts resulted in the inhibition of the activity, indicating that the observed activity is of aspartic protease nature and that cathepsin D could be matured in the AM despite the basic nature of its environment [44]. The identification of proteases, including cathepsins B, D, and L and aminopeptidases in the *R. prolixus* gut, has been associated solely with blood digestion machinery; however, these enzymes may achieve other roles. Indeed, it has been observed that cathepsin D gene expression increases in *Triatoma infestans* midgut during *T. cruzi* infection [45]. 

#### 3.2.4. Detoxification and Antioxidant Enzymes

Blood processing in the digestive tract of hematophagous insects results in the release of very high concentrations of heme, which may lead into iron-induced oxidative stress mediated by the Fenton reaction. To overcome this, triatomines have developed a number of strategies to eliminate these toxic blood products by heme crystallization into hemozoin, together with a wide range of antioxidant enzymes acting in the digestive tract [46,47]. We have identified in this work 40 and 37 antioxidant proteins implicated in detoxification in the AM and PM, respectively (Table S2), covering the whole panel of protection to face the ROS release and prevent damage that could be caused during blood digestion. This is in accordance with a previous study showing that *R. prolixus* has a unique and complex heme degradation pathway [48]. Hence, we have identified both Cu/Zn-superoxide dismutase (SOD, T1HRT6) and Mn-SOD (R4FJZ0), which are the first enzymes involved in the ROS detoxification process, by converting the superoxide anion (O_2_^.^) to hydrogen peroxide (H_2_O_2_) [49]. Analysis of the digestive tract transcriptome of *R. prolixus* enabled the identification of transcripts of three SODs in the AM: two cytosolic Cu/Zn-SOD and one mitochondrial Mn-SOD [14]. On the other hand, they identified five SOD isoforms in the PM: four cytosolic Cu/Zn-SOD and one mitochondrial Mn-SOD [14]. In addition to SOD, and amongst the most important enzymes implicated in redox metabolism and detoxification, we identified, in both the AM and PM, two catalases, four peroxidases, five glutaredoxin/thioredoxin, one glyoxalase, eight glutathione transferases, two sulfotransferases, and twelve dehydrogenases (Table S2), indicating the importance of this process. These enzymes are produced by *R. prolixus* to protect its tissues in response to the presence of heme released during blood processing [48,49,50] and/or to the presence of pathogens in the blood meal upon entry into the midgut, as observed in the case of the infection of *R. prolixus* by *Trypanosoma rangeli* [51] and *Triatoma infestans* following its infection by *T. cruzi* [45]. Amongst the antioxidant proteins involved in the detoxification processes, several proteins were not reported in the transcriptome of the *R. prolixus* digestive tract, such as the two catalases (T1HV37 and T1I0W4), which roles are to complement the action of SOD by the further decomposition of H_2_O_2_. Additional protection is provided by the *Rhodnius* heme binding protein (Q8T5U0), which preventive antioxidant role has been demonstrated in *R. prolixus* [49,52]. In fact, it controls the potentially deleterious reactivity of free heme and attenuates its toxicity by reducing its capacity to promote lipid peroxidation [48]. Surprisingly, we identified a single CYP-450 (T1I4Z9) in the AM and two CYP-450 (R4G3X7 and T1HUZ5) in the PM, while 15 and 17 different transcript isoforms were identified in the AM and PM transcriptomes, respectivly [14]. Comparing these findings to other antioxidant machinery identified by proteomic analyses of hematophagous insects show that *R. prolixus* has the most diversified and sophisticated antioxidant enzymatic machinery. Indeed, only 29 proteins implicated in the antioxidant process and detoxification were identified in *Ornithodoros erraticus* [22] and only thirteen in *Ornithodoros moubata* [21].

## 4. Conclusions

In this study, we reported the analysis of the midgut proteome of *R. prolixus* using high-throughput proteomic analysis tools. These data are complementary to the *R. prolixus* digestive tract transcriptomic data reported by Ribeiro et al., 2014 [14], and the 2-DE reference map of the AM of the insect [43]. The expression of several proteins for which transcripts are highly expressed in the midgut has been confirmed by our study at the protein scale, such as proteases and protease inhibitors. However, there is still a weak correlation between the transcriptome and the proteome midgut in terms of the number of expressed isoforms. This could be due to post-transcriptional and translational regulations and the difference in the half-life of mRNAs and protein turnovers [15]. It should not be forgotten that the proteome changes quickly over time, so small differences in timing could also explain the observed differences, as in this study, we combined the proteome of starved insects with those 6-h post blood-feeding, while the transcriptomic study was conducted on insects 12 h and up to five days post-feeding. Similarly to the transcriptome study, we have combined the proteomes of both starved and 6-h blood-fed insects. Contrary to the transcriptome experimental design, we chose to pool only these two conditions to further conduct a comparative study of the differentially expressed proteins, which is ongoing. Analysis of the midgut proteome of *R. prolixus* allowed the characterization of a very sophisticated hematophagic machinery, allowing both the ingestion and digestion of a large quantity of blood. We also showed the existence of proteolytic activity in the AM, ensured by cathepsin D. This suggests that these enzymes could be maturated in the AM. This enzymatic activity could also have other roles in AM, such as the interaction with *T. cruzi*. This high-throughput proteomic analysis could be a starting step for the selection of new targets for innovative control strategies of Chagas disease.

## Figures and Tables

**Table 1:** Protein functional classes and numbers belonging to blood ingestion and digestion machinery identified in *Rhodnius prolixus* anterior midgut (AM) and posterior midgut (PM) tissues.

Protein Class	Number of Proteins	
	**AM**	**PM**
Detoxification and antioxidant machinery	40	37
Proteases	27	29
Carbohydrates transport and metabolism	29	29
Lipids transport and metabolism	18	14
Heat Shock proteins	12	10
Lipocalins	11	13
Protease inhibitors	6	3
Immune	5	4

## Supplementary Materials

The following are available online at https://www.mdpi.com/2227-7382/8/3/16/s1, Figure S1: Proteolytic activity of *R. prolixus* AM under two different pH conditions. Table S1: *R. prolixus* midgut proteome. Table S2: Characterization of *R. prolixus* hematophagy machinery.

## References

[1] Barrozo R.B. (2019). Food recognition in hematophagous insects. Curr. Opin. Insect Sci..

[2] Hurd H. (2009). Parasite-Modified Vector Behavior. Encycl. Anim. Behav..

[3] Rassi A., Rassi A., Marin-neto J.A. (2010). Chagas disease. Lancet.

[4] World Health Organization (WHO) (2017). Chagas Disease Key Facts. https://www.who.int/news-room/fact-sheets/detail/chagas-disease-(american-trypanosomiasis).

[5] Domachowske J. (2019). Introduction to Clinical Infectious Diseases.

[6] López-vélez R., Norman F.F., Bern C. (2019). 103—American trypanosomiasis (Chagas Disease). Hunter’s Tropical Medicine and Emerging Infectious Diseases.

[7] Nunes-da-fonseca R., Berni M., Pane A., Araujo H.M. (2017). *Rhodnius prolixus*: From classical physiology to modern developmental biology. Genesis.

[8] De Fuentes-vicente J.A., Vidal-lópez D.G., Flores-villegas A.L., Moreno-rodríguez A., De Alba-alvarado M.C., Salazar-schettino P.M., Rodríguez-lópez M.H., Gutiérrez-cabrera A.E. (2019). Acta Tropica *Trypanosoma cruzi*: A review of biological and methodological factors in Mexican strains. Acta Trop..

[9] Azambuja P., Garcia E.S. (2005). *Trypanosoma rangeli* interactions within the vector *Rhodnius prolixus*—A mini review. Mem. Inst. Oswaldo Cruz.

[10] Guarneri A.A., Lorenzo M.G. (2017). Triatomine physiology in the context of trypanosome infection. J. Insect Physiol..

[11] Gonçalves C.S., Ávila A.R., De Souza W., Motta M.C.M., Cavalcanti D.P. (2018). Revisiting the *Trypanosoma cruzi* metacyclogenesis: Morphological and ultrastructural analyses during cell differentiation. Parasites Vectors.

[12] Kollien A.H., Schaub G.A. (2000). The Development of *Trypanosoma cruzi* in Triatominae. Parasitol. Today.

[13] Garcia E.S., Genta F.A., De Azambuja P., Schaub G.A. (2010). Interactions between intestinal compounds of triatomines and *Trypanosoma cruzi*. Trends Parasitol..

[14] Ribeiro J.M.C., Genta F.A., Sorgine M.H.F., Logullo R., Mesquita R.D., Paiva-Silva G.O., Majerowicz D., Medeiros M., Koerich L., Terra W.R. (2014). An Insight into the Transcriptome of the Digestive Tract of the Bloodsucking Bug, *Rhodnius prolixus*. PLoS Negl. Trop. Dis..

[15] Bevilacqua A., Ceriani M.C., Capaccioli S., Nicolin A. (2003). Post-transcriptional regulation of gene expression by degradation of messenger RNAs. J. Cell. Physiol..

[16] Burand J.P., Hunter W.B. (2013). RNAi: Future in insect management. J. Invertebr. Pathol..

[17] Mi H., Muruganujan A., Casagrande J.T., Thomas P.D. (2013). Large-scale gene function analysis with PANTHER Classification System. Nat. Protoc..

[18] Knight C.G., Barrett A.J. (1976). Interaction of human cathepsin D with the inhibitor pepstatin. Biochem. J..

[19] Mesquita R.D., Vionette-Amaral R.J., Lowenberger C., Rivera-Pomar R., Monteiro F.A., Minx P., Spieth J., Carvalho A.B., Panzera F., Lawson D. (2016). Erratum: Genome of *Rhodnius prolixus*, an insect vector of Chagas disease, reveals unique adaptations to hematophagy and parasite infection (Proceedings of the National Academy of Sciences of the United States of America (2015) 112 (14936–14941) DOI 10.107). Proc. Natl. Acad. Sci. USA.

[20] Geiger A., Soumana I.H., Tchicaya B., Rofidal V. (2015). Differential expression of midgut proteins in *Trypanosoma brucei gambiense*-stimulated vs. non-stimulated *Glossina palpalis gambiensis* flies. Front. Microbiol..

[21] Oleaga A., Obolo-Mvoulouga P., Manzano-Román R., Pérez-Sánchez R. (2017). A proteomic insight into the midgut proteome of *Ornithodoros moubata* females reveals novel information on blood digestion in argasid ticks. Parasites Vectors.

[22] Oleaga A., Obolo-Mvoulouga P., Manzano-Román R., Pérez-Sánchez R. (2015). Midgut proteome of an argasid tick, *Ornithodoros erraticus*: A comparison between unfed and engorged females. Parasites Vectors.

[23] Wood V., Lock A., Harris M.A., Rutherford K., Bähler J., Oliver S.G. (2019). Hidden in plain sight: What remains to be discovered in the eukaryotic proteome?. Open Biol..

[24] Feder M.E., Hofmann G.E. (1999). Heat-Shock Proteins, Molecular Chaperones, and the Stress Response: Evolutionary and Ecological Physiology. Annu. Rev. Physiol..

[25] Mahroof R., Kun Y.Z., Neven L., Subramanyam B., Bai J. (2005). Expression patterns of three heat shock protein 70 genes among developmental stages of the red flour beetle, *Tribolium castaneum* (Coleoptera: Tenebrionidae). Comp. Biochem. Physiol. A Mol. Integr. Physiol..

[26] Sanders H.R., Evans A.M., Ross L.S., Gill S.S. (2003). Blood meal induces global changes in midgut gene expression in the disease vector, *Aedes aegypti*. Insect Biochem. Mol. Biol..

[27] Kongsuwan K., Josh P., Zhu Y., Pearson R., Gough J., Colgrave M.L. (2010). Exploring the midgut proteome of partially fed female cattle tick (Rhipicephalus (Boophilus) microplus). J. Insect Physiol..

[28] Benoit J.B., Lopez-Martinez G., Patrick K.R., Phillips Z.P., Krause T.B., Denlinger D.L. (2011). Drinking a hot blood meal elicits a protective heat shock response in mosquitoes. Proc. Natl. Acad. Sci. USA.

[29] Paim R.M.M., Araujo R.N., Leis M., Sant’anna M.R.V., Gontijo N.F., Lazzari C.R., Pereira M.H. (2016). Functional evaluation of Heat Shock Proteins 70 (HSP70/HSC70) on *Rhodnius prolixus* (Hemiptera, Reduviidae) physiological responses associated with feeding and starvation. Insect Biochem. Mol. Biol..

[30] Ledizet M., Harrison L.M., Koski R.A., Cappello M. (2005). Discovery and pre-clinical development of antithrombotics from hematophagous invertebrates. Curr. Med. Chem. Cardiovasc. Hematol. Agents.

[31] Noeske-Jungblut C., Haendler B., Donner P., Alagon A., Possani L., Schleuning W.D. (1995). Triabin, a highly potent exosite inhibitor of thrombin. J. Biol. Chem..

[32] Campos I.T.N., Amino R., Sampaio C.A.M., Auerswald E.A., Friedrich T., Lemaire H.G., Schenkman S., Tanaka A.S. (2002). Infestin, a thrombin inhibitor presents in *Triatoma infestans* midgut, a Chagas’ disease vector: Gene cloning, expression and characterization of the inhibitor. Insect Biochem. Mol. Biol..

[33] Araujo R.N., Campos I.T.N., Tanaka A.S., Santos A., Gontijo N.F., Lehane M.J., Pereira M.H. (2007). Brasiliensin: A novel intestinal thrombin inhibitor from *Triatoma brasiliensis* (Hemiptera: Reduviidae) with an important role in blood intake. Int. J. Parasitol..

[34] van de Locht A., Lamba D., Bauer M., Huber R., Friedrich T., Kröger B., Höffken W., Bode W. (1995). Two heads are better than one: Crystal structure of the insect derived double domain Kazal inhibitor rhodniin in complex with thrombin. EMBO J..

[35] Cao J., Shi L., Zhou Y., Gao X., Zhang H., Gong H., Zhou J. (2013). Characterization of a new kunitz-type serine protease inhibitor from the hard tick Rhipicephalus hemaphysaloides. Arch. Insect Biochem. Physiol..

[36] Friedrich T., Kroger B., Bialojan S., Lemaire H.G., Hoffken H.W., Reuschenbach P., Otte M., Dodt J. (1993). A Kazal-type inhibitor with thrombin specificity from *Rhodnius prolixus*. J. Biol. Chem..

[37] Soares T.S., Buarque D.S., Queiroz B.R., Gomes C.M., Braz G.R.C., Araújo R.N., Pereira M.H., Guarneri A.A., Tanaka A.S. (2015). A Kazal-type inhibitor is modulated by Trypanosoma cruzi to control microbiota inside the anterior midgut of *Rhodnius prolixus*. Biochimie.

[38] Kollien A.H., Waniek P.J., Nisbet A.J., Billingsley P.F., Schaub G.A. (2004). Activity and sequence characterization of two cysteine proteases in the digestive tract of the reduviid bug *Triatoma infestans*. Insect Mol. Biol..

[39] Horn M., Caffrey C.R., Sojka D., Kopa P. (2013). New insights into the machinery of blood digestion by ticks. Trends Parasitol..

[40] Balczun C., Siemanowski J., Pausch J.K., Helling S., Marcus K., Stephan C., Meyer H.E., Schneider T., Cizmowski C., Oldenburg M. (2012). Intestinal aspartate proteases TiCatD and TiCatD2 of the haematophagous bug *Triatoma infestans* (Reduviidae): Sequence characterisation, expression pattern and characterisation of proteolytic activity. Insect Biochem. Mol. Biol..

[41] Terra W.R., Dias R.O., Ferreira C. (2019). Recruited lysosomal enzymes as major digestive enzymes in insects. Biochem. Soc. Trans..

[42] de Azambuja P., Guimarães J.A., Garcia E.S. (1983). Haemolytic factor from the crop of *Rhodnius prolixus:* Evidence and partial characterization. J. Insect Physiol..

[43] Vieira L.R., Polomé A., Mesquita R.D., Salmon D., Braz G.R.C., Bousbata S. (2015). Protein 2DE reference map of the anterior midgut of the blood-sucking bug *Rhodnius prolixus*. Proteomics.

[44] Barros V.C., Assumpção J.G., Cadete A.M., Santos V.C., Cavalcante R.R., Araújo R.N., Pereira M.H., Gontijo N.F. (2009). The role of salivary and intestinal complement system inhibitors in the midgut protection of Triatomines and mosquitoes. PLoS ONE.

[45] Buarque D.S., Braz G.R.C., Martins R.M., Tanaka-Azevedo A.M., Gomes C.M., Oliveira F.A.A., Schenkman S., Tanaka A.S. (2013). Differential Expression Profiles in the Midgut of *Triatoma infestans* Infected with *Trypanosoma cruzi*. PLoS ONE.

[46] Oliveira M.F., Silva J.R., Dansa-Petretski M., De Souza W., Lins U., Braga C.M.S., Masuda H., Oliveira P.L. (1999). Haem detoxification by an insect. Nature.

[47] Egan T.J. (2008). Haemozoin formation. Mol. Biochem. Parasitol..

[48] Paiva-Silva G.O., Cruz-Oliveira C., Nakayasu E.S., Maya-Monteiro C.M., Dunkov B.C., Masuda H., Almeida I.C., Oliveira P.L. (2006). A heme-degradation pathway in a blood-sucking insect. Proc. Natl. Acad. Sci. USA.

[49] Graça-Souza A.V., Maya-Monteiro C., Paiva-Silva G.O., Braz G.R.C., Paes M.C., Sorgine M.H.F., Oliveira M.F., Oliveira P.L. (2006). Adaptations against heme toxicity in blood-feeding arthropods. Insect Biochem. Mol. Biol..

[50] Paes M.C., Oliveira M.B., Oliveira P.L. (2001). Hydrogen peroxide detoxification in the midgut of the blood-sucking insect, *Rhodnius prolixus*. Arch. Insect Biochem. Physiol..

[51] Cosentino-Gomes D., Rocco-Machado N., Meyer-Fernandes J.R. (2014). *Rhodnius prolixus*: Modulation of antioxidant defenses by *Trypanosoma rangeli*. Exp. Parasitol..

[52] O’Donnell M. (2008). Insect Excretory Mechanisms.

