# Supplementary material for: High-Throughput Identification of the Rhodnius prolixus Midgut Proteome Unravels a Sophisticated Hematophagic Machinery
Source: Proteomes. 2020 Jul 24;8(3):16. doi: 10.3390/proteomes8030016 (PMC7564601; doi:10.3390/proteomes8030016)
Supplement: Supplementary file 1 [file proteomes-08-00016-s001.zip › Suppmementary files/Figure S1 Proteolytic activity.pptx]

## Slide 1
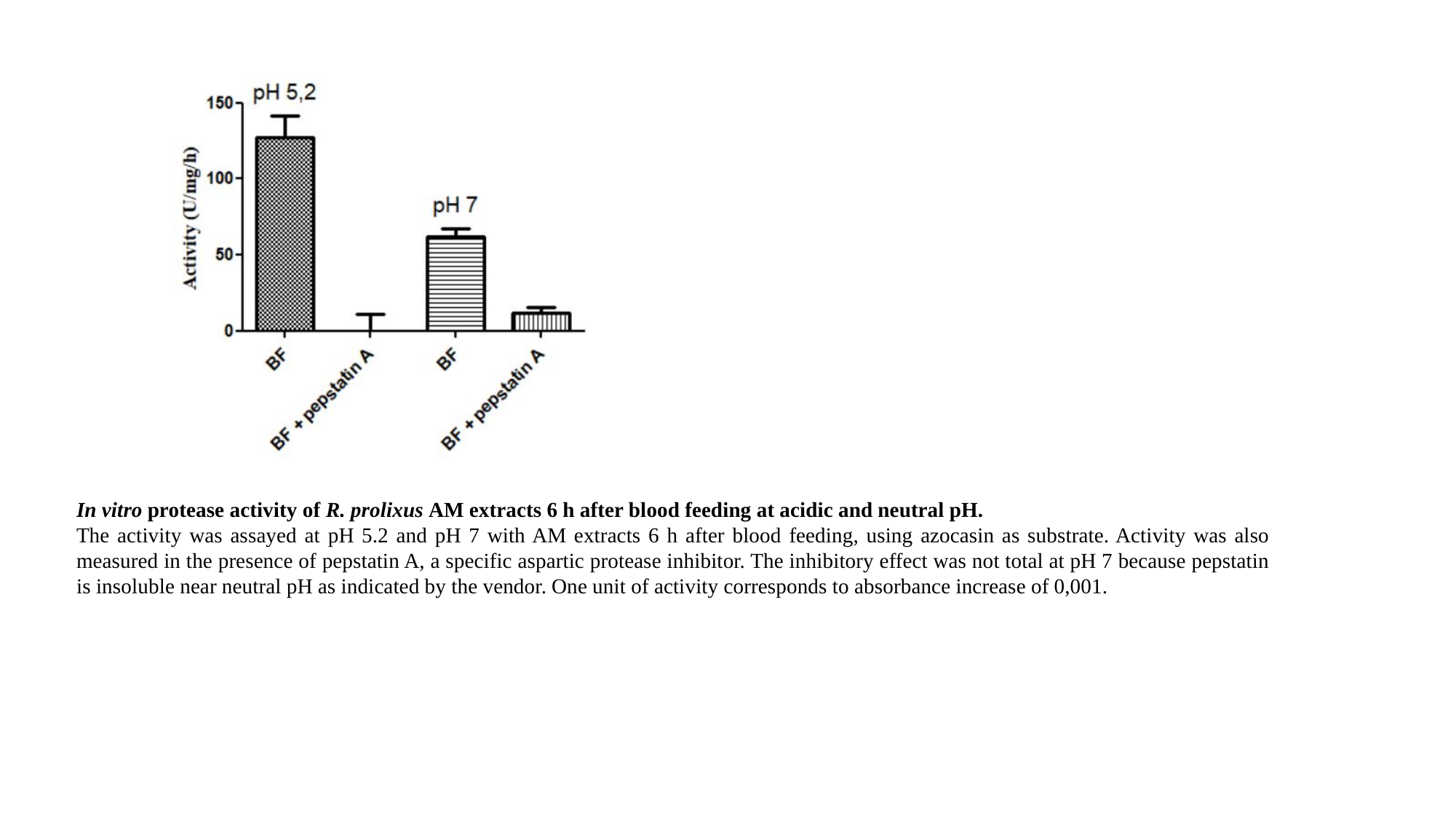

In vitro protease activity of R. prolixus AM extracts 6 h after blood feeding at acidic and neutral pH.
The activity was assayed at pH 5.2 and pH 7 with AM extracts 6 h after blood feeding, using azocasin as substrate. Activity was also measured in the presence of pepstatin A, a specific aspartic protease inhibitor. The inhibitory effect was not total at pH 7 because pepstatin is insoluble near neutral pH as indicated by the vendor. One unit of activity corresponds to absorbance increase of 0,001.
